# Health facility capacity to provide postabortion care in Afghanistan: a cross-sectional study

**DOI:** 10.1186/s12978-021-01204-w

**Published:** 2021-07-28

**Authors:** Farzana Maruf, Hannah Tappis, Enriquito Lu, Ghutai Sadeq Yaqubi, Jelle Stekelenburg, Thomas van den Akker

**Affiliations:** 1 Jhpiego Afghanistan, Kabul, Afghanistan; 2 Global Financing Facility, World Bank Group, Kabul, Afghanistan; 3 Athena Institute, Vrije Universitate, Amsterdam, The Netherlands; 4 Jhpiego, Baltimore, MD USA; 5 Ministry of Public Health, Kabul, Afghanistan; 6 University Medical Centre Groningen/University of Groningen, Groningen, The Netherlands; 7 Leeuwarden Medical Centre, Leeuwarden, The Netherlands; 8 Department of Obstetrics and Gynecology, Leiden University Medical Center, Leiden, The Netherlands

**Keywords:** Postabortion care, Medical treatment, Reproductive health, Capacity of health services, Afghanistan

## Abstract

**Background:**

Afghanistan has one of the highest burdens of maternal mortality in the world, estimated at 638 deaths per 100,000 live births in 2017. Infections, obstetric hemorrhage, and unsafe abortion are the three leading causes of maternal death. Contraceptive prevalence rate has fluctuated between 10 and 20% since 2006. The *2016 Afghanistan National Maternal and Newborn Health Quality of Care Assessment* evaluated facility readiness to provide quality routine and emergency obstetric and newborn care, including postabortion care services.

**Methods:**

Accessible public health facilities with at least five births per day (n = 77), a nationally representative sample of public health facilities with fewer than five births per day (n = 149), and 20 purposively selected private health facilities were assessed. Assessment components examining postabortion care included a facility inventory and record review tool to verify drug, supply, equipment, and facility record availability, and an interview tool to collect information on skilled birth attendants’ knowledge and perceptions.

**Results:**

Most facilities had supplies, equipment, and drugs to manage postabortion care, including family planning counseling and services provision. At public facilities, 36% of skilled birth attendants asked to name essential actions to address abortion complications mentioned manual vacuum aspiration (23% at private facilities); fewer than one-quarter mentioned counseling. When asked what information should be given to postabortion clients, 73% described family planning counseling need (70% at private facilities). Nearly all high-volume public health facilities with an average of five or more births per day and less than 5% of low volume public health facilities with an average of 0–4 deliveries per day reported removal of retained products of conception in the past 3 months. Among the 77 high volume facilities assessed, 58 (75%) reported using misoprostol for removal of retained products of conception, 59 (77%) reported using manual vacuum aspiration, and 67 (87%) reported using dilation and curettage.

**Conclusions:**

This study provides evidence that there is room for improvement in postabortion care services provision in Afghanistan health facilities including post abortion family planning. Access to high-quality postabortion care needs additional investments to improve providers’ knowledge and practice, availability of supplies and equipment.

**Supplementary Information:**

The online version contains supplementary material available at 10.1186/s12978-021-01204-w.

## Plain English summary

Afghanistan has one of the highest burdens of maternal mortality in the world. Infections, bleeding around childbirth, and unsafe abortion are the three leading causes of mortality in the country. The uptake of contraceptives is low, and only one-fifth of married women use contraceptives. A National Maternal and Newborn Health Quality of Care Assessment was conducted in 2016 at a selected number of public and private health facilities (n = 226; n = 20) to evaluate health facilities’ capacity to provide postabortion care, and skilled birth attendants’ knowledge and perceptions with regard to such care. Postabortion care is an essential package of services to make women survive complications of miscarriage and abortion and reduce unplanned pregnancies by providing postabortion family planning counseling and services, community empowerment, and mobilization. The result of this study showed that most facilities had supplies, equipment, and drugs to give postabortion care, including family planning services provision. However, there are gaps in birth attendants’ knowledge and their capacity to deliver high-quality postabortion care services at public and private facilities. This study provides evidence that there is room for improvement in postabortion care services provision at health facilities in Afghanistan. Access to high-quality postabortion care needs additional investments to improve providers’ knowledge and practice, and availability of supplies.

**Supplementary Information:**

The online version contains supplementary material available at 10.1186/s12978-021-01204-w.

## Background

Every year, an estimated 47,000 women die around the world because of unsafe abortion. Millions more face complications, including hemorrhage, infection, chronic pain, secondary infertility, and trauma to multiple organs [1]. Abortion-related complications are a preventable cause of mortality, accounting for almost 10% of maternal deaths that occurred over the past decades [2–4].

Afghanistan has one of the highest burdens of maternal mortality in the world, estimated at 638 deaths per 100,000 live births in 2017 [5], with infections, obstetric hemorrhage, and unsafe abortion as the three leading causes [6]. The contraceptive prevalence rate is low and has fluctuated between 10 and 20% since 2006; as of 2018, the total fertility rate was 5.1, and unmet need was at 25% [7]. Induced abortion is forbidden, except to save the life of a woman, according to the legal framework for safe abortion and postabortion care (PAC) in the country [8]. The Ministry of Public Health (MoPH) in Afghanistan has recognized treatment of incomplete abortion as an essential component of basic emergency obstetric and newborn care (BEmONC) since 2005. PAC includes both curative care (management of incomplete abortion and its complications) and preventive care (contraceptive counseling and services, and community mobilization). Both components are essential to ensure that high-quality care is received by women requiring these services [9, 10]. Providing high-quality PAC in all facilities is ethical and considered a humanitarian imperative; when high-quality PAC is available, morbidity and mortality associated with unsafe or incomplete abortion can be greatly reduced [11]. The World Health Organization (WHO) recommends use of manual vacuum aspiration (MVA) or medical treatment of abortions utilizing misoprostol-based regimens [12–14].

Afghanistan’s reproductive, maternal, newborn, child, and adolescent health strategy highlights the importance and scale of PAC services to be practiced by skilled birth attendants (SBAs; doctors and midwives) [15]. National clinical guidelines have recommended manual vacuum aspiration (MVA) for removal of retained products of conception since 2006, and clinical guidelines and in-service training packages for PAC including recommendations on use of misoprostol for treatment of miscarriage, were issued in 2017 [16]. However, data on implementation of PAC are scarce and the indicators for high-quality PAC services recommended by WHO are not included in Afghanistan’s health information system. The most recent study examining readiness to provide PAC services was completed in 2011 [17].

Studies evaluating abortion services in low- and middle-income countries have recommended making family planning services part of routine obstetrics and gynecology services to improve both postabortion and postpartum contraceptive provision. This includes ensuring supply of family planning commodities, and that service delivery points and protocols specifying family planning methods are offered at all service sites [18, 19]. Evidence shows that postabortion family planning is a high-impact practice that results in higher family planning uptake when it is provided proactively at the same time and location where a woman receives facility-based PAC [20, 21].

The purpose of this study was to assess the capacity of health facilities in Afghanistan to provide PAC services. This information is essential to frame policy discussions and strengthen evidence-based interventions to address gaps in provision of PAC services in the country.

## Methods

### Study design

The *2016 National Maternal and Newborn Health Quality of Care Assessment* was a cross-sectional health facility assessment assessing readiness to provide routine care and address major obstetric and newborn complications at 286 health facilities across all 34 provinces of Afghanistan. This paper presents a subset of findings related to health system capacity to provide PAC. Detailed methods for the overall assessment, data collection tools and analyses of other maternal and newborn health services are reported elsewhere [22].

### Sample

Facilities included all accessible public facilities with an average of five or more births per day between April 2015 and March 2016, as well as a representative sample of public facilities providing fewer than five births per day that were accessible at the time of the survey. The research team used probability proportional to size methods of cluster sampling to estimate results by facility type. Using a design effect of 1.5% to account for clustering of providers within facilities, the team estimated that a total of 147 public health facilities (HFs) would need to be assessed to estimate results with 10% precision, 95% confidence: four district hospitals (DHs), 30 comprehensive health centers (CHCs), 61 basic health centers (BHCs), 43 sub-health centers (SHCs), and 11 family health houses (FHHs). Twenty private-sector facilities were purposively sampled to provide a snapshot of private facilities in all regions of the country. This sample is not statistically representative for all private-sector facilities [22].

### Data collection

Two data collection methods were used to assess capacity for PAC service provision: a facility inventory and record review tool to verify availability of drugs, supplies, and equipment, infrastructure, and facility records, and an interview tool to collect information on SBAs’ knowledge, practices and perceptions. Tool content was adapted from the Demographic and Health Survey Service Provision Assessment, and from emergency obstetric and newborn care (EmONC) assessments supported by the Averting Maternal Death and Disability program [23–25]. See Additional file 1: Table S1 for additional details on data collection tool content and alignment with national guidelines and training packages.

Data collection at HFs with an average of at least five deliveries per day was completed between May 14, and August 3, 2016. Data collection at HFs that averaged fewer than five deliveries per day was completed from November 5, 2016, to January 5, 2017. Each team consisted of three data collectors (female midwives and doctors) and aimed to complete data collection within 2 to 3 days per facility.

Facility readiness for PAC was defined as availability of equipment, supplies, provider’s knowledge, and documentation at the point of care.

Data collection was carried out using CommCare software installed on Android tablets, allowing for logic and consistency checks, quality control, and online submission of the data to the central database [22].

### Analysis

For analysis purposes, facility types were defined as follows: (1) specialized hospital (SH), regional hospital (RH), and provincial hospital (PH); (2) DHs with an average of five or more deliveries per day; (3) DHs and CHCs with an average of fewer than five deliveries per day; and (4) BHCs, SHCs, FHHs, and other primary health care facilities. These classifications reflect levels of care with service delivery expectations outlined in Afghanistan’s *Essential Package of Hospital Services* (for SH, RH and PH) and *Basic Package of Health Services* (for DH, CHC, BHC, SHC); all are expected to provide care 24 h/day, 7 days/week, including treatment of incomplete miscarriage/abortion using manual vacuum aspiration [26, 27].

Descriptive statistics were used for analysis. A Chi-squared test was used to test for differences in facility readiness and routine care practices by public facility type. Tests of association for analyses of provider knowledge were adjusted for clustering of providers within a facility. All statistical analyses were conducted using Stata. Data from private facilities are based on purposive sampling and are not intended for direct comparison compared with the public health facilities.

## Results

Data collection teams visited a total of 226 public health facilities (37 SH, RH and PH; 30 DH with an average of 5 or more deliveries per day [high volume DH], 37 DH with less than 5 deliveries per day [low volume DH], 114 BHC, SHC or FHH) and 20 private health facilities. Interviews were conducted with a total of 721 SBAs, including 333 health providers at SH, RH and PH; 228 at high-volume DHs; 69 at low-volume DHs and CHCs; 104 at BHCs, SHCs, and FHHs; and 64 at private HFs.

### Health facility characteristics (Table 1)

**Table 1:** Characteristics of health facilities

Characteristics of health facilities	Facility type						
	Provincial, regional, and specialty hospitals (n = 37)	District hospitals with 5 or more deliveries per day (n = 40)	District hospitals and comprehensive health centers with 0–4 deliveries per day (n = 37)	Basic health centers, sub-health centers, and family health houses (n = 112)	p-value	All public sector (n = 226)	Private hospitals (n = 20)
Facilities that provide 24-h coverage for delivery of services (EmONC)	100.0% (37)	100.0% (40)	56.8% (21)	41.1% (46)	< 0.001	63.7% (144)	90.0% (18)
Facility manager reports that misoprostol is used for treatment of incomplete abortion	75.7% (28)	75.0% (30)	2.7% (1)	3.6% (4)	< 0.001	27.9% (63)	40.0% (8)
Facility manager reports that manual vacuum aspiration has been used for removal of retained products of conception in the past 3 months	75.7% (28)	77.5% (31)	0.0% (0)	4.5% (5)	< 0.001	28.3% (64)	10.0% (2)
Facility manager reports dilation and curettage or evacuation and curettage have been used for removal of retained products of conception in the past 3 months	97.3% (36)	80.0% (32)	2.7% (1)	0.0% (0)	< 0.001	30.5% (69)	65% (13)
Recordkeeping							
Abortion complications (hemorrhage and/or sepsis)							
Percentage of facilities with register available for review	72.5%	73.0%	8.0%	18.6%		47.3%	45.0%
Average number of months with data recorded	9	7	3	4		5	4
Average number of cases recorded per month	29	8	0	1	< 0.001	10	4
Postabortion women discharged with a contraceptive method							
Percentage of facilities with register available for review	64.9%	57.5%	21.6%	33.0%		40.7%	30.0%
Average number of months with data recorded	7	6	3	4		5	4
Average number of cases recorded per month	1	1	0	0	0.136	1	0

In 64 of 226 public HFs (28%), facility management reported performing MVA for removal of retained product of conception in the last 3 months. A higher proportion of SHs/RHs/PHs (76%) and high-volume DHs (78%) reported performing MVA for removal of retained product of conception compared to other facilities with significantly lower proportions. A similar proportion (63 of 226, 28%) and distribution of facilities reported using misoprostol for treatment of incomplete abortion.

In 69 of 226 public HFs (31%), facility management reported performing sharp curettage to remove retained product of conception in the last 3 months. A higher proportion of SHs/RHs/PHs (97%) and high-volume DHs (80%) performed sharp curettage, compared to other facility types with less than 5% of facilities using this method to remove retained product of conception.

Of 226 public HFs, 107 (47%) reported having a register to record abortion-related complications. Availability of a register also differed significantly across facility types, with a higher proportion of SHs/RHs/PHs (73%) and high-volume DHs (73%) having a register than low-volume DHs/CHCs (8%) and BHCs/SHCs/FHHs (19%). Around 41% (n = 92) public HFs reported having a register to track postabortion women discharged with a method of contraception from HFs. Facility logbooks recorded an average of 29 cases of postabortion complications per month and very low rates of women discharged with postabortion contraception at all health facilities, with an average of only one woman per month at SHs, RHS, and PHs.

### Availability of supplies, equipment, drugs, and guidelines (Table 2)

**Table 2:** Availability of supplies, equipment, drugs, and guidelines for provision of postabortion care services

Available items [percentage (number)]	Facility type						
	Provincial, regional, and specialty hospitals (n = 37)	District hospitals with 5 or more deliveries per day (n = 40)	District hospitals and comprehensive health centers with 0–4 deliveries per day (n = 37)	Basic health centers, sub-health centers, and family health houses (n = 112)*	p-value	All public sector (n = 226)	Private facilities (n = 20)
Functional manual vacuum aspirator and cannula	75.7% (28)	82.5% (33)	67.6% (25)	64.3% (72)	0.267	69.9% (158)	75.0% (15)
Dilation and curettage kit	89.2% (33)	85.0% (34)	59.5% (22)	60.7% (68)	0.004	69.5% (157)	85.0% (17)
Misoprostol	56.8% (21)	52.5% (21)	13.5% (5)	8.9% (10)	< 0.001	25.2% (57)	60.0% (12)
Guidelines or national treatment protocol for emergency obstetric and newborn care	43.2% (16)	47.5% (19)	54.1% (20)	42.0% (47)	0.214	45.1% (102)	35.0% (7)
Guidelines for the pre-referral management of major obstetric and newborn complications	32.4% (12)	32.5% (13)	54.1% (20)	40.2% (45)	0.350	39.8% (90)	50.0% (10)
Family planning commodities							
Male condoms	81.1% (30)	90.0% (36)	86.5% (32)	85.7% (96)	0.737	85.8% (194)	65.0% (13)
Female condoms	8.1% (3)	0.0% (0)	5.4% (2)	7.1% (8)	0.087	5.8% (13)	5.0% (1)
Oral contraceptive pills	91.9% (34)	95.0% (38)	91.9% (34)	87.5% (98)	0.916	90.3% (204)	65.0% (13)
Intrauterine devices	86.5% (32)	92.5% (37)	89.2% (33)	86.6% (97)	0.859	88.1% (199)	75.0% (15)
Implants	16.2% (6)	7.5% (3)	8.1% (3)	2.7% (3)	0.002	6.6% (15)	15.0% (3)
Injectable hormones (e.g., Depo-Provera)	86.5% (32)	92.5% (37)	78.4% (29)	74.1% (83)	0.286	80.1% (181)	55.0% (11)
Emergency contraception pill packets	54.1% (20)	37.5% (15)	37.8% (14)	35.7% (40)	0.586	39.4% (89)	25.0% (5)

Of 226 public HFs, 158 (70%) had functional MVA and cannula in the delivery room. A dilation and curettage (D&C) kit was available in 157 (70%) of 226 public health facilities. A significantly higher proportion of PHs/RHs/SHs (53%) and high-volume DHs (53%) had misoprostol in the delivery room compared to other facility types. Fewer than half of the public HFs (102 of 226; 45%) had EmONC guidelines and protocols available in the delivery rooms.

More than 80% of public HFs had male condoms, oral contraceptive pills, intrauterine devices, and injectables available. Emergency contraceptive pills and subdermal contraceptive implants were available in around 40% and 7% of facilities sampled, respectively.

### Skilled birth attendant knowledge on postabortion care (Table 3)

**Table 3:** Skilled birth attendant knowledge on postabortion care

[Percentage (number)]	Facility type						
	Provincial, regional, and specialty hospitals (n = 315)	District hospitals with 5 or more deliveries per day (n = 233)	District hospitals and comprehensive health centers with 0–4 deliveries per day (n = 69)	Basic health centers, sub-health centers, and family health houses (n = 104)	p-value	All public sector (n = 721)	Private facilities (n = 69)
Reported having received any training on basic emergency obstetric and newborn care in the past 3 years	27.0% (90)	18.0% (42)	21.7% (15)	25.0% (26)	0.129	24.0% (173)	23.2% (16)
Knowledge of actions to take when presented with a woman with complications from incomplete abortion							
Assess vaginal bleeding	59.0% (186)	54.1% (126)	56.5% (39)	55.8% (58)	< 0.001	56.7% (409)	66.7% (46)
Assess vital signs	54.9% (173)	52.4% (122)	50.7% (35)	57.7% (60)	< 0.001	54.1% (390)	62.3% (43)
Begin IV fluids	74.3% (234)	70.8% (165)	73.9% (51)	67.3% (70)	< 0.001	72.1% (520)	78.3% (54)
Begin antibiotics	38.4% (121)	40.8% (95)	36.2% (25)	31.7% (33)	< 0.001	38.0% (274)	36.2% (25)
Do (manual/electric) vacuum aspiration	31.7% (100)	39.1% (91)	42.0% (29)	36.5% (38)	< 0.001	35.8% (258)	23.2% (16)
Provide counseling	22.5% (71)	30.5% (71)	15.9% (11)	22.1% (23)	< 0.001	24.4% (176)	15.9% (11)
Refer	11.4% (36)	18.0% (42)	26.1% (18)	32.7% (34)	< 0.001	18.0% (130)	8.7% (6)
Knowledge of counseling to provide a woman being treated for an incomplete abortion							
Information on how to prevent reproductive tract infection/HIV	30.5% (96)	33.0% (77)	34.8% (24)	36.5% (38)	< 0.001	32.6% (235)	31.9% (22)
Information about when a woman can conceive again	43.8% (138)	48.1% (112)	27.5% (19)	45.2% (47)	< 0.001	43.8% (316)	44.9% (31)
Counseling on family planning and services	73.3% (231)	70.0% (163)	84.1% (58)	77.9% (81)	< 0.001	73.9% (533)	69.6% (48)
Refer for family planning methods	54.0% (170)	56.2% (131)	49.3% (34)	59.6% (62)	< 0.001	55.1% (397)	52.2% (36)
Information on social support	27.0% (85)	29.2% (68)	27.5% (19)	22.1% (23)	< 0.001	27.0% (195)	15.9% (11)
Information about the consequences of an unsafe abortion	34.9% (110)	37.3% (87)	20.3% (14)	21.2% (22)	< 0.001	32.3% (233)	33.3% (23)
Does a woman have the right to choose a family planning method? (Level of agreement with the statement: a woman should not choose a family planning method until she consults with her husband)							
Strongly agree	35.6% (112)	42.9% (100)	27.5% (19)	17.3% (18)	< 0.001	34.5% (249)	26.1% (18)
Agree	34.9% (110)	30.5% (71)	60.9% (42)	57.7% (60)		39.3% (283)	53.6% (37)
Neither agree nor disagree (neutral)	7.0% (22)	4.7% (11)	4.3% (3)	3.8% (4)		5.5% (40)	5.8% (4)
Disagree	7.0% (22)	6.4% (15)	7.2% (5)	16.3% (17)		8.2% (59)	5.8% (4)
Strongly disagree	0.0% (0)	0.4% (1)	0.0% (0)	1.9% (2)		0.4% (3)	8.7% (6)
Level of agreement with the statement: a woman who has not had a boy child should not be encouraged to use family planning							
Strongly agree	23.8% (75)	25.8% (60)	14.5% (10)	11.5% (12)	< 0.001	21.8% (157)	10.1% (7)
Agree	19.0% (60)	21.5% (50)	20.3% (14)	30.8% (32)		21.6% (156)	37.7% (26)
Neither agree nor disagree (neutral)	11.1% (35)	14.6% (34)	4.3% (3)	6.7% (7)		11.0% (79)	11.6% (8)
Disagree	25.4% (80)	20.2% (47)	59.4% (41)	44.2% (46)		29.7% (214)	29.0% (20)
Strongly disagree	4.1% (13)	2.1% (5)	1.4% (1)	3.8% (4)		3.2% (23)	4.3% (3)

Of 721 SBAs interviewed, 258 (36%) asked to name essential actions to address complications of abortion mentioned MVA, 520 (72%) indicated IV fluid, and more than half of SBAs mentioned checking vital signs (390, or 54%) and assessing for vaginal bleeding (409, or 57%). Counseling was listed by fewer than one-quarter (24%) of SBAs interviewed. When asked what information should be given to a woman being treated for an incomplete abortion, 533 SBAs (74%) described the need for counseling on family planning, 397 (55%) mentioned the need for referral on family planning methods and 310 (44%) noted the need for counseling on when a woman could conceive again. Fewer than one-third mentioned information on social support, consequences of unsafe abortion, and infection prevention. More than two-thirds of SBAs (74%) interviewed believed that a woman should not choose a family planning method until she consults with her husband, and fewer than half of SBAs (43%) interviewed believed that a woman who has not had a boy child should not be encouraged to use family planning.

### Snapshot of private health facility capacity

Eight of 20 facilities (40%) reported using misoprostol for treatment of incomplete abortions, and two facilities (10%) reported using MVA for treatment of incomplete abortions in the last 3 months. Thirteen of 20 facilities (65%) reported SBAs ever performed sharp curettage in the last 3 months. Private facilities recorded an average of four postabortion complications in a month, while six of 20 (30%) facilities had registers to record postabortion women when discharged from facility with a contraceptive method. Twelve of 20 HFs (60%) had misoprostol in the delivery rooms. Of the 20 health facilities assessed, 15 (75%) and 17 (85%) had functional MVA and a D&C kit, respectively. More than 50% of facilities had male condoms, oral contraceptive pills, intrauterine devices, and injectables, and implants were available in 15% of private facilities. Only 16 of 69 SBAs (23%) asked to name essential actions to address complications of abortion mentioned MVA. When asked what information should be given to PAC clients, 48 of 69 SBAs (70%) described the need for counseling on family planning. More than two-thirds of SBAs interviewed (80%) reported believing that a woman should not choose a family planning method until she consults with her husband, and nearly half of SBAs interviewed (48%) reported believing that a woman who has not had a boy child should not use family planning methods.

## Discussion

Our study reveals gaps in availability of supplies, equipment, and drugs for PAC at all levels of the health system in 2016. EmONC guidelines were available at fewer than half of public and private health facilities. These guidelines indicate managing incomplete abortions and miscarriage using MVA as a signal function but lack the medical treatment of incomplete abortion with misoprostol and family planning component of PAC. Studies show substantial promise to extend the option for treating incomplete abortion and miscarriage with misoprostol at the district and lower levels in remote, rural, and impoverished areas, where access to PAC and surgery is mostly restricted [13, 28, 29]. Programs should ensure that both MVA and misoprostol are available at HFs, and women should be informed of appropriate treatment options, staff must be trained, and communities should be informed [20].

Our study found that misoprostol was available in more than half of public and private health facilities in Afghanistan. This is not surprising as misoprostol is included on the Essential Medicines List for prevention and management of postpartum hemorrhage, however its use for PAC is not specified. In 2017, the year following this assessment, the national standards and training package for PAC were updated to include misoprostol for managing complications of abortion in addition to MVA [16]. These updates were already underway before findings from our study were disseminated, however findings highlighted the work needed to ensure widespread roll-out and uptake of the policy and protocol changes. It is important for health managers to ensure that the updated national standards and training package for PAC are available at health facilities and describe revised practices that are important instruments for ensuring quality, and treatment with misoprostol is included at both in-service and pre-service curricula [20, 30, 31].

Our study also found that D&C procedure is still widely practiced in Afghanistan, despite MVA being the method recommended for treatment of incomplete abortion in national guidelines. Routine D&C is riskier and more painful for removal of retained products of conception; it is no longer recommended by WHO and should be phased out gradually [16].

This study revealed gaps in SBAs’ knowledge and capacity to deliver high-quality PAC services at both public and private health facilities—mainly on use of MVA, providing family planning counseling to women, starting IV fluids, checking vital signs, and assessing for bleeding. The gaps documented in providers’ knowledge are consistent with the results of the 2010 EmONC assessment findings in Afghanistan. Recommendations emerging from this study were not implemented enough to obtain substantial results, and they need more investment and innovative approaches to be implemented holistically [17]. Specifically, SBAs’ knowledge and capacity should be improved through evidence-based and cost-effective capacity-building approaches, such as short, periodic, additional “skills and drills” sessions or “fire drills,” that have the potential to help ensure knowledge and skills are retained over time [32].

Most of the facilities visited had a mix of short- and long-term contraceptive commodities available at the time of the assessment. Contraception and safe abortion care go hand in hand in the strategy to reduce unwanted pregnancies, unsafe abortions, and maternal deaths. Increasing access to modern contraception and timely provision of family planning services are essential components to reducing unmet need, unintended or unwanted pregnancies, and the abortions or unplanned births that often follow, and provision of counseling to women should be voluntary, confidential, nondirective and respectful [18, 21, 33]. However, social norms and health care providers’ personal beliefs could be factors in the low levels of family planning counseling and method acceptance. Some factors that challenge the effective implementation of PAC programs include provider bias and/or resistance to provide family planning to postabortion clients due to abortion-related stigma and cultural barriers, and women who are disempowered to make decisions regarding contraceptive use. Studies show that women lacked the authority to make family planning decisions without involvement of men in some settings. In many societies, extreme pressure is placed on woman to bear sons, including in Afghanistan; they may encounter violence, abandonment, or stigma for birthing girls instead of boys. Therefore, engaging men and boys in the process of transforming attitudes around inequality in gender norms is critical.

Evidence suggests the importance of timely education on PAC and postabortion contraception, and involving women, men, and health care providers [34–37].

This study also shows major gaps in documentation of postabortion cases at public and private health facilities. Documentation for PAC services could be further improved to capture information on postabortion clients receiving contraceptives by method before leaving the facility and at return visits, and the signs and symptoms of postabortion complications that would need medical attention. Health management information systems should include indicators on provision and quality of PAC services as an essential measure of program and provider performance and accountability [38, 39].

This study had certain limitations. It was conducted in 2016 and does not provide current picture of PAC services in Afghanistan, or extent to which new protocols have been rolled out. It was designed to assess the quality of broader maternal and newborn health services, so some critical aspects of PAC, such as providers’ actual performance on the job, particularly how well they counsel and interact with patients, were not captured. Information on community mobilization and awareness, an important component of PAC, was not in the scope of this assessment. Data collection on health services was based on verbal information of facility managers, not documentation of services or actual triangulation of available data sources at various levels. The private-sector snapshot did not represent the overall picture of PAC in private HFs in Afghanistan and cannot be compared with nationally representative data from public health facilities. Although the snapshot captured by this assessment suggests that the private sector’s capacity to provide PAC services might be similar to the public sector’s, there is a need for accurate, informative, and generalizable data on the capacity of private health facilities that deliver high-quality PAC considering compliance with current protocols on medical treatment of incomplete abortion with misoprostol. Despite these limitations, this study provides important information on the capacity of the public and private health system in Afghanistan to deliver PAC around the time that updated guidelines and training materials were issued, and provides insight on needs for strengthening health services to save women’s lives.

## Conclusions

High-quality PAC can lead to reductions in the levels of abortion-related morbidity and mortality. Access to high-quality curative services, particularly at the lower level of services, where BEmONC is expected to be in place, still needs additional investment to improve skills, support performance of providers, and ensure availability of supplies and medicine, including liming the risker routine practices like D&C. Misoprostol provides great promise for improving access to safe PAC services, especially where provision of surgical means is not feasible, and it is critical that the government should legitimize the availability of misoprostol in the Essential Medicines List for medical treatment purposes.

This study also noted certain gaps on preventive aspects of PAC, particularly access to counseling and contraception, and it is imperative to provide high-quality postabortion family planning counseling universally. Policymakers and other stakeholders should address the underlying gaps in implementation of a comprehensive PAC program and improve the documentation and reporting of data to drive accountability for progress. Achieving further progress requires rigorous commitment for reproductive and sexual rights, sound public health programming, and a multisectoral approach with regards to achieving Sustainable Development Goals in Afghanistan.

## Supplementary Information

**Additional file 1:** **Table S1.** Indicators of facility readiness to provide post-abortion care included in *2016 National Maternal and Newborn Health Quality of Care Assessment* tools, and alignment with national guidelines and training packages.

## Data Availability

Data are available from the MoPH upon request. Requests should be directed to the MoPH’s Evaluation and Health Information Systems Department (ehis.moph@gmail.com).

## Abbreviations

BEmONC: Basic emergency obstetric and newborn care
BHC: Basic health center
CHC: Comprehensive health center
D&C: Dilation and curettage
DH: District hospital
EmONC: Emergency obstetric and newborn care
FHH: Family health house
MoPH: Ministry of Public Health
MVA: Manual vacuum aspiration
PAC: Postabortion care
PH: Provincial hospital
RH: Regional hospital
SBA: Skilled birth attendant
SH: Specialized hospital
SHC: Sub-health center
WHO: World Health Organization

## References

[1] Postabortion Care (PAC) Consortium. Imagining a world free from postabortion care stigma. http://www.september28.org/2017/.

[2] Say L, Chou D, Gemmill A, Tuncalp O, Moller AB, Daniels J, Gulmezoglu AM, Temmerman M, Alkema L (2014). Global causes of maternal death: a WHO systematic analysis. Lancet Glob Health.

[3] Department of Reproductive Health and Research, World Health Organization (WHO) (2011). Unsafe abortion: global and regional estimates of the incidence of unsafe abortion and associated mortality in 2008.

[4] Starbird E, Norton M, Marcus R (2016). Investing in family planning: key to achieving the sustainable development goals. Glob Health Sci Pract.

[5] World Health Organization. Trends in maternal mortality 2000 to 2017: estimates by WHO, UNICEF, UNFPA, World Bank Group and the United Nations Population Division. Geneva: World Health Organization; 2019. https://apps.who.int/iris/handle/10665/327596.

[6] KIT Royal Tropical Institute, Afghanistan National Statistics and Information Authority (2019). Afghanistan health survey 2018.

[7] World Bank Group, MoPH. Options for strengthening family planning in basic package of health services in Afghanistan; 2019.

[8] The World’s Abortion Laws. https://reproductiverights.org/worldabortionlaws?country=AFG.

[9] Safe Abortion. Technical and policy guidance for health systems second edition technical and policy guidance for health systems. https://www.who.int/reproductivehealth/en/.

[10] Owolabi OO, Biddlecom A, Whitehead HS (2019). Health systems’ capacity to provide post-abortion care: a multicountry analysis using signal functions. Lancet Glob Health.

[11] Kulczycki A (2016). The imperative to expand provision, access and use of misoprostol for post-abortion care in sub-Saharan Africa. Afr J Reprod Health.

[12] WHO (2015). Health worker roles in providing safe abortion care and post-abortion contraception.

[13] Edelman A, Mark A (2017). Medical abortion reference guide: induced abortion and postabortion care at or after 13 weeks gestation ('second trimester').

[14] WHO (2011). WHO model list of essential medicines: 17th list.

[15] Afghanistan Ministry of Public Health (MoPH) (2017). Afghanistan reproductive, maternal, newborn, child and adolescent health strategy: 2017–2021.

[16] Ministry of Public Health Deputy Minister Office for Health Care Services Provision Reproductive, Maternal, Newborn, Child and Adolescent Health Directorate: Post Abortion Care National Clinical Service Guideline, 2017. Kabul, Afghanistan

[17] Ansari N, Zainullah P, Kim YM, Tappis H, Kols A, Currie S, Haver J, van Roosmalen J, Broerse JE, Stekelenburg J (2015). Assessing post-abortion care in health facilities in Afghanistan: a cross-sectional study. BMC Pregnancy Childbirth.

[18] Biswas KK, Pearson E, Shahidullah SM, Sultana S, Chowdhury R, Andersen KL (2017). Integrating postabortion care, menstrual regulation and family planning services in Bangladesh: a pre-post evaluation. Reprod Health.

[19] Huntington D, Hassan EO, Attallah N, Toubia N, Naguib M, Nawar L (1995). Improving the medical care and counseling of postabortion patients in Egypt. Stud Fam Plann.

[20] Huber D, Curtis C, Irani L, Pappa S, Arrington L (2016). Postabortion care: 20 years of strong evidence on emergency treatment, family planning, and other programming components. Glob Health Sci Pract.

[21] High Impact Practices in Family Planning (2019). Family planning high impact practices list.

[22] Jhpiego (2017). Afghanistan national maternal and newborn health quality of care assessment 2016: key findings report.

[23] Bartlett L, Cantor D, Lynam P, Kaur G, Rawlins B, Ricca J, Tripathi V, Rosen HE, Quality of Maternal and Newborn Care Study Group of the Maternal and Child Health Integrated Program (2015). Facility-based active management of the third stage of labour: assessment of quality in six countries in sub-Saharan Africa. Bull World Health Organ.

[24] The DHS Program: Service Provision Assessments (SPA). https://dhsprogram.com/What-We-Do/Survey-Types/SPA.cfm.

[25] Averting Maternal Death and Disability (AMDD). https://www.mailman.columbia.edu/research/averting-maternal-death-and-disability-amdd0.

[26] Ministry of Public Health. A basic package of health services for Afghanistan, Revised July 2010. Kabul, Afghanistan.

[27] Ministry of Public Health. The essential package of hospital services for Afghanistan, 2015. Kabul, Afghanistan.

[28] Perehudoff K, Pizzarossa LB, Stekelenburg J (2018). Realising the right to sexual and reproductive health: access to essential medicines for medical abortion as a core obligation. BMC Int Health Hum Rights.

[29] Cleeve A, Byamugisha J, Gemzell-Danielsson K, Tumwesigye NM, Atuhairwe S, Faxelid E (2016). Women’s acceptability of misoprostol treatment for incomplete abortion by midwives and physicians—secondary outcome analysis from a randomized controlled equivalence trial at district level in Uganda. PLoS ONE.

[30] Tang J, Kapp N, Dragoman M, De Souza JP (2013). WHO recommendations for misoprostol use for obstetric and gynecologic indications. Int J Gynaecol Obstet.

[31] Makenzius M, Oguttu M, Klingberg-Allvin M, Gemzell-Danielsson K, Odero TMA, Faxelid E (2017). Post-abortion care with misoprostol-equally effective, safe and accepted when administered by midwives compared to physicians: a randomised controlled equivalence trial in a low-resource setting in Kenya. BMJ Open.

[32] Ameh CA, White S, Dickinson F, Mdegela M, Madaj B, van den Broek N (2018). Retention of knowledge and skills after Emergency Obstetric Care training: a multicountry longitudinal study. PLoS ONE.

[33] High Impact Practices in Family Planning (2019). Postabortion family planning: a critical component of postabortion care.

[34] Mosha I, Ruben R, Kakoko D (2013). Family planning decisions, perceptions and gender dynamics among couples in Mwanza, Tanzania: a qualitative study. BMC Public Health.

[35] Rehnström Loi U, Otieno B, Oguttu M, Gemzell-Danielsson K, Klingberg-Allvin M, Faxelid E (2019). Abortion and contraceptive use stigma: a cross-sectional study of attitudes and beliefs in secondary school students in western Kenya. Sex Reprod Health Matters.

[36] United Nations Population Fund (UNFPA) (2019). Unfinished business: the pursuit of rights and choices for all.

[37] Berry-Bibee EN, St Jean CJ, Nickerson NM, Haddad LB, Alcime MM, Lathrop EH (2018). Self-managed abortion in urban Haiti: a mixed-methods study. BMJ Sex Reprod Health.

[38] Jain A, Aruldas K, Mozumdar A, Tobey E, Acharya R (2019). Validation of two quality of care measures: results from a longitudinal study of reversible contraceptive users in India. Stud Fam Plann.

[39] Sedgh G, Singh S, Shah IH, Ahman E, Henshaw SK, Bankole A (2012). Induced abortion: incidence and trends worldwide from 1995 to 2008. Lancet.

