# Supplementary material for: Health facility capacity to provide postabortion care in Afghanistan: a cross-sectional study
Source: Reprod Health. 2021 Jul 28;18:160. doi: 10.1186/s12978-021-01204-w (PMC8317397; doi:10.1186/s12978-021-01204-w)
Supplement: Supplementary file 1 — Additional file 1: Table S1. Indicators of facility readiness to provide post-abortion care included in 2016 National Maternal and Newborn Health Quality of Care Assessment tools, and alignment with national guidelines and training packages. [file 12978_2021_1204_MOESM1_ESM.docx]

**Table S1: Indicators of facility readiness to provide post-abortion care included in *2016 National Maternal and Newborn Health Quality of Care Assessment* tools, and alignment with national guidelines and training packages**

|  | **Corresponding data collection tool (Question)*** | **Notes on alignment of data collection tool content with national guidelines and training packages** |
| --- | --- | --- |
| **Facility readiness** | | |
| Guidelines or national treatment protocol for emergency obstetric and newborn care | Tool A (376) | Emergency obstetric and newborn care guidelines and treatment protocols include content related to removal of retained products of conception. At the time of study design, PAC guidelines had not yet been launched. |
| Guidelines for the pre-referral management of major obstetric and newborn complications | Tool A (377) | It was assumed that if guidelines for pre-referral management of major obstetric complications were in place, these would include guidelines for pre-referral management of miscarriage and abortion-related complications. |
| Functional manual vacuum aspirator and cannula | Tool A (311) | Recommended method for uterine evacuation at time of assessment |
| Dilation and curettage kit | Tool A (312) | Emergency obstetric and newborn care guidelines (2006) include D&C but this procedure is no longer recommended by WHO |
| Misoprostol | Tool A (307) | Not yet included in national guidelines and treatment protocols at time of assessment but expected to be widely available for other obstetric indications. PAC guidelines including medical treatment of abortions were issued in 2017. |
| Male condoms | Tool A (502) | As noted above, emergency obstetric care guidelines include guidance on managing incomplete abortions and miscarriage using MVA as a signal function but lack the medical treatment of incomplete abortion with misoprostol and family planning component of PAC. PAC guidelines including family planning counseling and service provision were issued in 2017. |
| Female condoms |  |  |
| Oral contraceptive pills |  |  |
| Intrauterine devices |  |  |
| Implants |  |  |
| Injectable hormones (e.g., Depo-Provera) |  |  |
| Emergency contraception pill packets |  |  |
| **Provider readiness** | | |
| ***Provider knowledge*** | | |
| Reported having received any training on basic emergency obstetric and newborn care in the past 3 years | Tool B (B119) | It was assumed that training on basic emergency obstetric care would include training on management of complications of abortion |
| Knowledge of actions to take when presented with a woman with complications from incomplete abortion | Tool B (211) | MVA was the recommended method of uterine evacuation at the time of the assessment. PAC guidelines including medical treatment of abortions were issued in 2017. |
| Assess vaginal bleeding |  |  |
| Assess vital signs |  |  |
| Begin IV fluids |  |  |
| Begin antibiotics |  |  |
| Do (manual/electric) vacuum aspiration |  |  |
| Provide counseling |  |  |
| Refer |  |  |
| Knowledge of counseling to provide a woman being treated for an incomplete abortion: | Tool B (212) | Emergency obstetric and newborn care guidelines (2006) do not include detailed guidance on counseling for PAC clients. PAC guidelines including counseling on family planning and social support were issued in 2017. |
| Information on how to prevent reproductive tract infection/HIV |  |  |
| Information about when a woman can conceive again |  |  |
| Counseling on family planning and services |  |  |
| Refer for family planning methods |  |  |
| Information on social support |  |  |
| Information about the consequences of an unsafe abortion |  |  |
| ***Provider skills*** | | |
| Provider skills | *Not assessed* |  |
| ***Provider beliefs and confidence*** | | |
| Does a woman have the right to choose a family planning method? (Level of agreement with the statement: A woman should not choose a family planning method until she consults with her husband.) | Tool B (411) | National guidelines on family planning service provision address women’s rights to choose a family planning method, but as noted above, emergency obstetric and newborn care guidelines (2006) do not include detailed guidance on counseling for PAC clients. PAC guidelines including counseling on family planning (including women’s right to choose a method) were issued in 2017. |
| Strongly agree |  |  |
| Agree |  |  |
| Neither agree nor disagree (neutral) |  |  |
| Disagree |  |  |
| Strongly disagree |  |  |
| Provider confidence in providing PAC | *Not assessed* |  |
| **Available at https://figshare.com/articles/online_resource/Afghanistan_National_Maternal_and_Newborn_Health_Quality_of_Care_Assessment_2016/12363974/1* | | |
